# Long‐term outcome after fetal endoscopic tracheal occlusion for congenital diaphragmatic hernia: systematic review

**DOI:** 10.1002/uog.70302

**Published:** 2026-08-29

**Authors:** S. Shah, R. Ruiz Roman, K. H. Nicolaides

**Affiliations:** ^1^ GKT School of Medical Education, King's College London London UK; ^2^ Fetal Medicine Research Institute, King's College Hospital London UK

**Keywords:** congenital anomaly, congenital diaphragmatic hernia, fetal surgery, fetoscopy, neonatal outcome

## Abstract

**Objective:**

This systematic review aimed to evaluate the long‐term outcomes of infants who had undergone fetal endoscopic tracheal occlusion (FETO) for congenital diaphragmatic hernia (CDH).

**Methods:**

PubMed, MEDLINE, EMBASE and the Cochrane Central Register of Controlled Trials (CENTRAL) were searched from inception to October 2025 for studies reporting infant outcome ≥ 1 year post FETO in the following domains: growth and nutritional, neurodevelopmental, audiological, cardiac, respiratory, gastrointestinal and musculoskeletal. Quality was assessed using the revised Cochrane risk‐of‐bias tool for randomized trials and the Newcastle–Ottawa scale for cohort studies. Meta‐analysis was not performed due to outcome heterogeneity and a narrative synthesis was conducted instead.

**Results:**

Fourteen studies (one randomized controlled trial and 13 cohort studies), including a total of 395 infants, were analyzed. The duration of follow‐up ranged from 7 to 87 months. Among domains of morbidity, respiratory complications were the most prevalent, although most improved over time. Growth failure affected a significant proportion of infants at 1 year, but considerable catch‐up growth was demonstrated by 2 years. Gastrointestinal morbidity was common, with gastroesophageal reflux disease reported in 25–50% of cases and hernia recurrence in 21–43%. Neurodevelopmental outcome was generally reassuring. Multiple studies found no significant differences in long‐term outcome between infants that underwent FETO and those that did not after adjusting for disease severity.

**Conclusion:**

FETO‐treated CDH survivors experience substantial but improving multisystem morbidity that may be driven by factors independent of the intervention itself, emphasizing the need for long‐term multidisciplinary follow‐up. © 2026 The Author(s). *Ultrasound in Obstetrics & Gynecology* published by John Wiley & Sons Ltd on behalf of International Society of Ultrasound in Obstetrics and Gynecology.

## Introduction

Congenital diaphragmatic hernia (CDH) is one of the most common life‐threatening birth defects, with an incidence between 1 in 2000 and 1 in 5000^1^. It is characterized by herniation of the abdominal viscera into the thoracic cavity, which leads to pulmonary hypoplasia. Treatment has shifted from open fetal surgery to percutaneous fetal endoscopic tracheal occlusion (FETO)^2^. FETO is a reversible prenatal intervention in which an endotracheal balloon is placed via a trocar through the umbilicus of the mother^3^. By occluding temporarily the fetal trachea, FETO promotes lung expansion through retained lung fluid, aiming to reduce pulmonary hypoplasia and improve postnatal survival.

Despite advances in prenatal diagnosis and neonatal care, CDH remains associated with substantial mortality^4^. The Tracheal Occlusion To Accelerate Lung growth (TOTAL) trials are landmark randomized controlled trials (RCTs) that have provided the strongest evidence to date for the efficacy of FETO^5^, ^6^, but they reported infant outcome for only 6 months postnatally. As survival rates improve, there is growing recognition of the need for comprehensive, multidisciplinary long‐term follow‐up care. However, data on long‐term outcome after FETO are limited. Most existing studies focus primarily on survival or short‐term neonatal outcome, with long‐term outcome often reported inconsistently, descriptively or as a secondary endpoint. The fetal medicine community has emphasized that current evidence, while demonstrating survival benefit, remains insufficient to recommend FETO as the standard of care^7^, particularly given the lack of long‐term morbidity data and the significant risk of prematurity.

The aim of this systematic review was to assess the long‐term outcome of infants who had undergone FETO for CDH and were followed up for at least 1 year postoperatively.

## Methods

### Eligibility criteria and search strategy

This systematic review was conducted and reported in accordance with the Preferred Reporting Items for Systematic reviews and Meta‐Analyses (PRISMA) guideline^8^. It was registered on the International Prospective Register of Systematic Reviews (PROSPERO) database (registration number: CRD420251178676) on 19 November 2025. No ethical approval or informed consent was required for this systematic review, as it analyzed only anonymized, aggregate data from published studies. The population was fetuses with CDH, the exposure was FETO and the primary outcomes were long‐term infant outcomes in the following domains: growth and nutritional, neurodevelopmental, audiological, cardiac, respiratory, gastrointestinal and musculoskeletal. We included RCTs and observational cohort studies in the English language that reported postnatal follow‐up for ≥ 1 year post FETO. Animal studies, Letters to the Editor, case reports, case series containing < 10 cases and studies which did not report long‐term outcome separately for FETO‐treated patients were excluded from the narrative synthesis.

PubMed, MEDLINE and EMBASE via Ovid, and the Cochrane Central Register of Controlled Trials (CENTRAL) were searched from inception until October 2025 to retrieve eligible studies. While Scopus was not included in the search strategy, EMBASE was used as a comprehensive biomedical database. We utilized keywords and medical subject heading (MeSH) terms for each database, connected by Boolean operators. Filters were applied to limit the search to human studies in the English language and to exclude preprints. The full search strategy is provided in Appendix S1. The search was carried out by one author (S.S.) and checked by another author (R.R.R.).

### Screening of eligible studies

References were managed using Zotero, and Rayyan software was used for study screening and removal of duplicates. Two reviewers (S.S., R.R.R.) independently performed title and abstract screening, with decisions masked to one another. Disagreements were resolved through discussion until consensus was reached. Full‐text screening was conducted by one reviewer (S.S.) and checked by a second reviewer (R.R.R.), with any discrepancies resolved by discussion. Although case reports and case series with < 10 cases were excluded from the formal narrative synthesis, they were reviewed separately to identify rare complications^1^, ^9^, ^10^.

### Data extraction and quality assessment

Data extraction was conducted by one reviewer (S.S.), with inconsistencies checked by a second reviewer (R.R.R.). The following data were extracted from each study: first author; year of publication; country; study design; number of infants that underwent FETO; infant sex; gestational age at which FETO was performed, at birth and at which the occluding device was removed; birth weight; initial lung‐to‐head ratio (LHR) (i.e. at diagnosis or first prenatal assessment); type, severity and laterality of CDH; duration of postoperative follow‐up; and long‐term infant outcomes in the abovementioned domains. Observed‐to‐expected LHR was derived primarily from ultrasonography, although some studies incorporated magnetic resonance imaging‐based measurements. Where studies reported baseline characteristics or outcomes as percentages within subgroups (e.g. survivors only), we extracted these as reported. Where possible, we recorded the size of the subgroup; thus, denominators may differ from the total cohort size reported for that study. Several studies reported baseline characteristics without stratification by FETO exposure, limiting the extraction of FETO‐specific data. We identified two instances of overlapping cohorts; in these cases, data from all relevant reports were used for outcome description, but participants were counted only once when calculating the overall sample size.

Study quality was assessed by one reviewer (S.S.) and verified by a second reviewer (R.R.R.). The quality of studies was assessed using the revised Cochrane risk‐of‐bias tool for randomized trials (RoB 2) and the Newcastle–Ottawa scale (NOS) for cohort studies. Studies evaluated using the RoB 2 tool were classified as having a high risk of bias, some concerns or a low risk of bias, according to established guidance. Although the NOS does not provide official quality thresholds, we followed the categorization used in previous reviews: a total score of 0–4 indicated poor quality, 5–7 indicated fair quality and 8–9 indicated high quality^11^.

### Data synthesis

When comparative effect estimates were reported by original studies, we extracted and reported them; otherwise, outcomes were summarized descriptively. Data were extracted as proportions or percentages of affected patients, mean ± SD or median (range), depending on how the results were presented originally. Long‐term outcomes across all domains were primarily descriptive. There was substantial heterogeneity in protocols, populations, follow‐up duration and outcome definitions. Given the substantial clinical and methodological heterogeneity across studies, quantitative meta‐analysis was not considered appropriate and results were synthesized qualitatively. In many instances, the available data were not reported in a format that permitted reliable quantitative pooling, and conducting a meta‐analysis would have risked producing misleading summary estimates. Formal assessment of reporting bias was not undertaken because quantitative synthesis was not performed and the number of included studies was small, inviting the possibility of unreliability. Similarly, a formal certainty‐of‐evidence assessment was not conducted because outcomes were synthesized qualitatively, effect estimates were not pooled, and outcome heterogeneity limited the applicability of certainty frameworks, such as the GRADE framework.

## Results

### Study selection

The literature search identified 1064 studies from four databases (Figure 1). Of those, 551 records were duplicates and were therefore excluded before screening. Of the remaining 513 studies, 37 were considered potentially eligible after title and abstract screening. Following full‐text review, 14 studies were ultimately included in the systematic review. Several studies that otherwise met the inclusion criteria were excluded at full‐text review because the outcomes were not reported separately for the FETO group. Excluded studies are summarized in Table S1.

**Figure 1:**
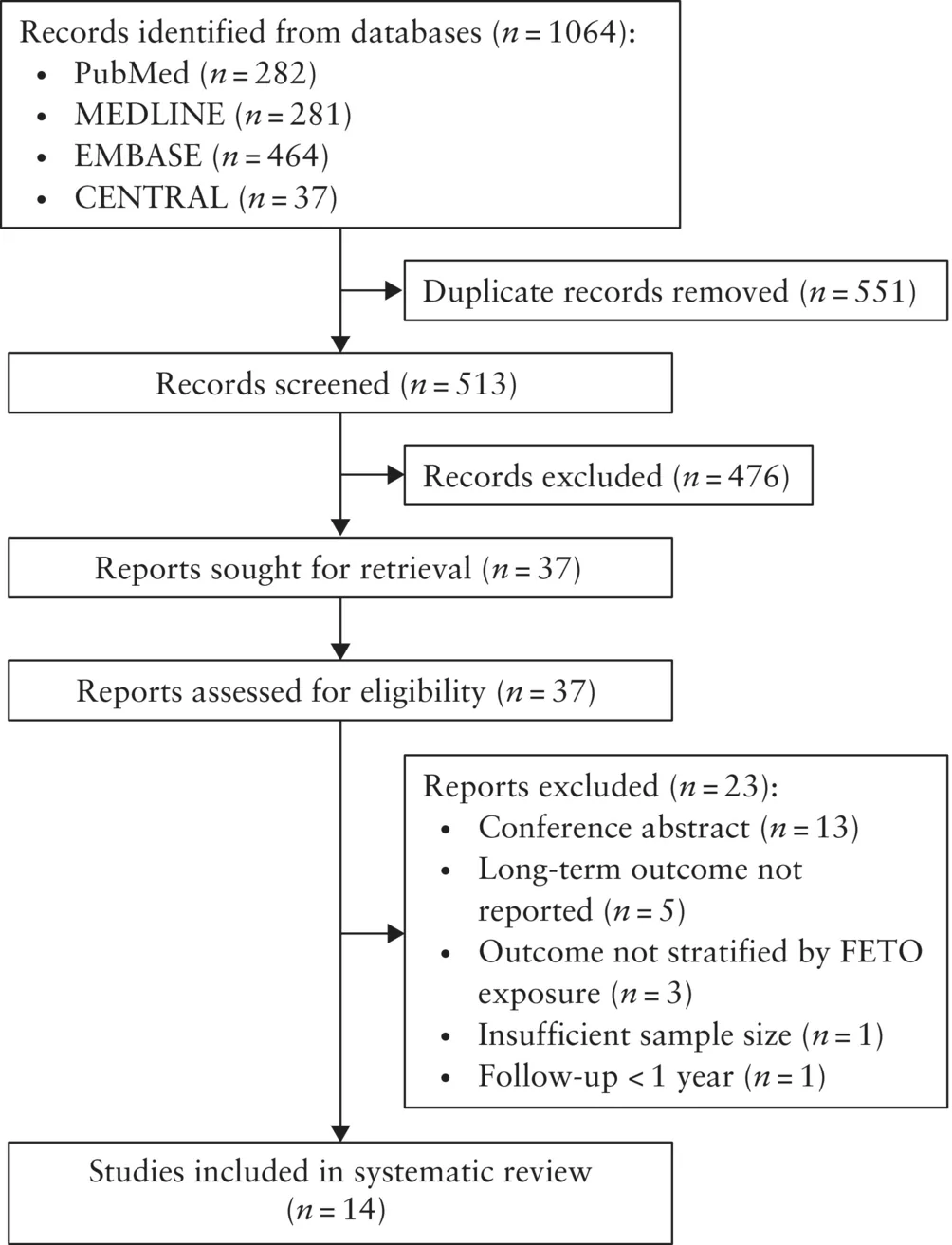
Flowchart summarizing inclusion of studies in systematic review. CENTRAL, Cochrane Central Register of Controlled Trials; FETO, fetal endoscopic tracheal occlusion.

### Study characteristics

Among the 14 studies included in the systematic review was one RCT and 13 cohort studies^12^, ^13^, ^14^, ^15^, ^16^, ^17^, ^18^, ^19^, ^20^, ^21^, ^22^, ^23^, ^24^, ^25^. Study characteristics are summarized in Table 1. Together, these studies included a total of 395 distinct CDH cases that underwent FETO. The number of infants included in individual studies ranged from 10 to 106. The duration of follow‐up varied considerably across studies, ranging from 7 to 87 months, with several studies reporting follow‐up at multiple timepoints. Most studies focused on severe CDH, with both left‐sided and right‐sided defects represented. Where reported, the majority of cases had left‐sided CDH. Imaging modalities used in the included studies are detailed in Table S2.

**Table 1:** Characteristics of 14 studies included in systematic review assessing long‐term outcomes after fetal endoscopic tracheal occlusion (FETO) for congenital diaphragmatic hernia (CDH)

Study	Country	Study design	FETO cases *(n)*	Male fetal sex (%)	GA at FETO (weeks)	BW (g)	GA at birth (weeks)	Initial raw LHR or O/E‐LHR (%)	Severity of CDH	Laterality of CDH	GA at removal of occluding device (weeks)	Follow‐up duration (months)
Harrison (2003)^20^	USA	Cohort	19	NA	27.4 ± 1.6	Survivors *vs* non‐survivors: 1870 ± 553 *vs* 1345 ± 606	NA§	Survivors *vs* non‐survivors: 0.92 ± 0.19 *vs* 0.81 ± 0.30	Severe	16 left‐sided, 3 right‐sided	At delivery	41 (26–63)
Cortes (2005)^12^	USA	RCT	11	86	30.8 ± 2.0	1610 ± 380	31.1 ± 1.7	1.06 ± 0.15	Severe	11 left‐sided	At delivery	12, 24**
Deprest (2005)^23^	Belgium, UK, Spain	Cohort	20	NA	26	NA	33.2 (27–38)	0.7 (0.4–0.9)	Severe	13 left‐sided, 7 right‐sided	34	19 (7–26)
Ali (2013)^13^	UK	Cohort	61	56	23–32	2240 (550–3800)	34 (24–40)	0.6 (0.3–1.2)	NA	NA	33–37	12–60
Zani (2014)^22^	UK	Cohort	41	61	NA	2510 (1356–3820)	35 (31–40)	NA	NA	NA	NA	36 (12–87)
Belfort (2017)^24^	USA	Cohort	11	NA	27.9 ± 1.1	2600 (1500–3100)	35.3 ± 2.2	0.85 (0.70–1.00)	Severe	11 left‐sided	34.1 ± 1.1	12, 24**
Morandi (2018)^17^	Italy	Cohort	30	NA	28.3 ± 1.37	2575 ± 583†	35.5 ± 2.8	31.3 ± 6.9†	Severe	8 left‐sided, 5 right‐sided*	34 ± 1.5	12, 24, 36**
Ali (2019)^21^	UK	Cohort	106	NA	23–32	NA	NA	NA	Severe	91 left‐sided, 15 right‐sided	NA	≥ 12
Style (2019)^18^	USA	Cohort	19	62.5*	28.4 (25.7–29.7)	NA	35.3 (32.9–37.0)	27 (21–32)	Mild, moderate and severe	≥ 16 left‐sided¶	34.3 (31.9–35.1)	12**
Macchini (2020)^25^	Italy	Cohort	10	50	26–31	2600 ± 485	35.7 ± 2.5	30.6 ± 5.6	Severe	6 left‐sided, 4 right‐sided	34	18 (9–26)
Sferra (2023)^14^	USA	Cohort	18	56	28 ± 5 (27 ± 3 to 29 ± 6)	3000 (2000–3000)	38 (35–39)	23 (18–25)	Severe (Type C or D)	16 left‐sided, 2 right‐sided	33 ± 4	69.6 (24–84)
Pertierra Cortada (2023)^15^	Spain, USA	Cohort	32	43.8†	NA	2638 (2266–2902)	36.62 (35.10–38.20)	NA	Moderate and severe	29 left‐sided, 3 right‐sided	NA	≥ 24
Dütemeyer (2024)^19^	Germany, Belgium, France, Italy	Cohort	47	43	NA	NA‡	35.14 (33.14 − 37.21)	NA	Severe	47 left‐sided	NA	12, 24, 72**
Sferra (2024)^16^	USA	Cohort	18	56	28 ± 5 (27 ± 3 to 29 ± 6)	2889 (2300–3100)	38 (35–39)	23 (18–25)	Severe (Type C or D)	16 left‐sided, 2 right‐sided	33 ± 4	12, > 24**

Only first author is given for each study. Data are presented as mean ± SD, median, range or median (range), unless specified otherwise. Sferra *et al*.^14^ and Sferra *et al*.^16^ report same cohort (*n* = 18) at different follow‐up timepoints. Ali *et al*.^13^ (*n* = 61) and Ali *et al*.^21^ (*n* = 106) represent overlapping cohorts from same institution. Not all outcomes were reported at all follow‐up timepoints.

* Among severe CDH cases only.

† Among survivors only.

‡ Reported only as *Z*‐score. §Individual data only.

¶ Exact count not reported; subgroup analysis suggests vast majority of cases were left‐sided.

** Data are given as timepoints at follow‐up, rather than duration of follow‐up. BW, birth weight; GA, gestational age; LHR, lung‐to‐heart ratio; NA, not available; O/E, observed‐to‐expected; RCT, randomized controlled trial.

### Risk of bias and quality assessment

The RCT included in this systematic review had an overall high risk of bias according to the RoB 2 tool (Table 2), primarily due to participants and carers being aware of their assigned intervention during the trial due to the nature of FETO. Among the 13 cohort studies, eight were deemed to be of fair quality and five were deemed to be of high quality, according to the NOS (Table 3). Most cohort studies lacked comparability of the exposed and unexposed cohorts.

**Table 2:** Quality assessment of randomized controlled trial using revised Cochrane risk‐of‐bias tool for randomized trials (RoB 2)

Study	Bias arising from randomization process	Bias due to deviation from intended intervention	Bias due to missing outcome data	Bias in measurement of outcome	Bias in selection of reported result	Overall
Cortes (2005)^12^	Low	High	Low	Low	Some concerns	High

Only first author is given.

**Table 3:** Quality assessment of cohort studies using Newcastle–Ottawa scale

	Selection					Outcome				
Study	Representativeness of exposed cohort	Selection of unexposed cohort	Ascertainment of exposure	Outcome not present at start	Comparability of cohorts	Assessment	Follow‐up length	Adequacy of follow‐up	Total	Quality
Harrison (2003)^20^	★	★	★	★	—	★	★	★	7/9	Fair
Deprest (2005)^23^	★	★	★	★	★	★	★	★	8/9	High
Ali (2013)^13^	★	—	★	★	★	★	★	★	7/9	Fair
Zani (2014)^22^	★	★	★	★	★	★	★	—	7/9	Fair
Belfort (2017)^24^	★	★	★	★	★	★	★	★	8/9	High
Morandi (2018)^17^	★	★	★	★	—	★	★	★	7/9	Fair
Ali (2019)^21^	★	★	★	★	—	★	★	★	7/9	Fair
Style (2019)^18^	★	★	★	★	★★	★	★	—	8/9	High
Macchini (2020)^25^	★	★	★	★	★	★	★	★	8/9	High
Sferra (2023)^14^	★	★	★	★	★	★	★	★	8/9	High
Pertierra Cortada (2023)^15^	★	★	★	★	★	★	★	—	7/9	Fair
Dütemeyer (2024)^19^	★	—	★	★	★	★	★	—	6/9	Fair
Sferra (2024)^16^	★	★	★	★	★	★	★	—	7/9	Fair

Only first author is given for each study. Maximum of one star available for each item, with exception of comparability, for which maximum of two stars were available.

### Growth and nutritional outcomes

Growth and nutritional outcomes were reported in five studies (one RCT^12^ and four cohort studies^13^, ^14^, ^15^, ^19^), with follow‐up ranging from 12 to 84 months (Table S3). Growth impairment and feeding difficulties were prevalent among infants treated with FETO, particularly in early childhood. For instance, growth failure affected approximately 25% of survivors in one cohort and reliance on enteral feeding was reported in up to two‐thirds of patients in another cohort^13^, ^14^. However, considerable catch‐up growth was observed at later follow‐up^12^. Pertierra Cortada *et al*.^15^ found no significant association between failure to thrive at 2 years and either prenatal disease severity or FETO treatment status.

### Neurodevelopmental outcome

Neurodevelopmental outcome was reported in seven studies (one RCT^12^ and six cohort studies^13^, ^14^, ^15^, ^16^, ^19^, ^23^), with follow‐up ranging from 7 to 84 months (Table S3). Overall, most studies reported reassuring neurodevelopmental outcome, with low rates of clinically significant impairment. Formal neurodevelopmental assessment was reported in only one study, using the Capute Scales (Clinical Linguistic and Auditory Milestone Scales (CLAMS) and Cognitive Adaptive Test (CAT))^16^. Median CLAMS and CAT developmental quotient (DQ) scores improved between 12 and > 24 months, suggesting neurodevelopmental improvement over time, although a proportion of infants remained at risk for neurodevelopmental delay at the 12‐month assessment.

### Audiological outcome

Audiological outcome was reported in two studies (one RCT^12^ and one cohort study^13^), with follow‐up ranging from 12 to 60 months (Table S3). Across both studies, a minority of infants developed hearing impairment, however a larger number of infants required hearing amplification at follow‐up. Importantly, the RCT that compared FETO‐exposed and unexposed groups did not report consistent differences in audiological outcome.

### Cardiac outcome

Cardiac outcome was reported in five studies (one RCT^12^ and four cohort studies^17^, ^18^, ^19^, ^24^), with follow‐up ranging from 12 to 72 months (Table S3). The studies focused primarily on pulmonary hypertension, cardiac function and need for extracorporeal membrane oxygenation (ECMO). ECMO utilization was consistently higher in non‐FETO cohorts. In addition, rates of persistent pulmonary hypertension at 12 months post FETO were low^17^, ^18^, ^19^.

### Respiratory outcome

Respiratory outcome was reported in 11 studies (one RCT^12^ and 10 cohort studies^13^, ^14^, ^15^, ^17^, ^19^, ^20^, ^21^, ^22^, ^24^, ^25^), with follow‐up ranging from 9 to 87 months (Table S3). Long‐term respiratory morbidity was reported frequently and included airway complications, ongoing respiratory support and chronic pulmonary symptoms. Cortes *et al*.^12^ reported that respiratory support requirements were common at discharge and during early follow‐up but declined over time, with progressively fewer children requiring oxygen supplementation over 2 years of follow‐up. Chronic respiratory symptoms, including reactive airway disease, chronic cough and upper airway symptoms, were reported across cohorts. Imaging‐based studies demonstrated larger tracheal diameters in infants who underwent FETO compared with those who did not, particularly in the early postnatal period; however, tracheal growth trajectories and measures of pulmonary function were similar between exposed and unexposed groups.

### Gastrointestinal outcome

Gastrointestinal outcome was reported in eight studies (one RCT^12^ and seven cohort studies^13^, ^14^, ^15^, ^20^, ^21^, ^22^, ^25^), with follow‐up ranging from 9 to 87 months (Table S3). Overall, gastrointestinal morbidity was common among FETO‐treated survivors; however, the majority of reported disease was managed medically rather than surgically. Gastroesophageal reflux disease was the most frequently reported outcome, with the reported prevalence varying across studies, ranging from approximately 25% to 50%. A substantial proportion of infants required antireflux medications during follow‐up, while a smaller subset required surgical intervention for reflux^13^. In one cohort, fundoplication was reported in 9–20% of patients, according to the laterality of CDH^21^. Recurrent diaphragmatic hernia was also reported, with recurrence rates ranging from approximately 21% to 43%. Notably, recurrence rates varied according to anatomical factors, with higher rates in right‐sided CDH compared with left‐sided defects.

### Musculoskeletal outcome

Musculoskeletal outcome was reported in three cohort studies^13^, ^21^, ^22^, with follow‐up ranging from 12 to 87 months (Table S3). Among these studies, musculoskeletal abnormality was reported frequently. Thoracic, spinal and chest wall deformities, including pectus excavatum, pectus carinatum and thoracic asymmetry, were present.

Overall, respiratory and gastrointestinal outcomes were supported by relatively more consistent and objective reporting, whereas neurodevelopmental and musculoskeletal outcomes were limited by heterogeneity and reliance on non‐standardized assessments.

## Discussion

### Interpretation of results

This systematic review evaluated long‐term outcomes and multidomain morbidity in 395 infants who had undergone FETO for CDH across one RCT and 13 cohort studies. Overall, survivors demonstrated long‐term morbidity in multiple domains, particularly growth and nutritional, respiratory and gastrointestinal outcomes. It was difficult to disentangle the impact on long‐term outcome of fetal intervention from that of confounding variables, such as baseline disease severity, prematurity, gestational age at FETO and laterality of the defect. Where reported, right‐sided CDH was associated with a higher recurrence rate compared to left‐sided defects, although data were limited and were reported inconsistently across studies. Importantly, multiple cohort studies reported no significant differences in long‐term outcome between exposed and unexposed groups, even after adjustment for prenatal disease severity, supporting the view that FETO may not independently modify longer‐term morbidity trajectory.

The most prevalent long‐term complication was respiratory morbidity, reflecting the pulmonary hypoplasia intrinsic to CDH. Although widened tracheal diameters were observed more frequently following FETO, this was not associated with worse respiratory function or increased morbidity compared with non‐FETO cohorts^17^, ^22^. In addition, Pertierra Cortada *et al*.^15^ suggested that growth impairment could be associated more closely with underlying disease burden than with exposure to prenatal therapy. Likewise, long‐term gastrointestinal outcome may reflect anatomical disruption to a greater extent than it does fetal intervention. Case reports and small case series, which were excluded from the formal narrative synthesis and reviewed separately, predominantly described long‐term airway complications, such as tracheomegaly and tracheomalacia. There were also isolated reports of periocular injury and infants thriving without postnatal morbidity^1^, ^9^, ^10^. Importantly, serial evaluations within a multidisciplinary clinic demonstrated progressive improvement in disease morbidity^14^. These findings align with previous reviews demonstrating that FETO improves survival in severe CDH but does not eliminate long‐term morbidity^26^, ^27^, ^28^. Our review extends this understanding by providing comprehensive assessment across multiple outcome domains beyond the neonatal period.

Notably, many survivors included in the studies synthesized herein belonged to the highest prenatal risk categories, which were associated historically with near‐universal mortality, making survival itself a clinically meaningful outcome despite persistent morbidity. With only one RCT and substantial methodological heterogeneity between studies, our findings underscore the need for standardized severity definitions, consistent outcome reporting and long‐term multidisciplinary follow‐up to better define the role of FETO in modifying long‐term outcome of CDH.

### Strengths and limitations

This review has several strengths. It was conducted according to a preregistered protocol, followed PRISMA reporting guidance and applied predefined inclusion criteria and domain‐based outcome categorization. Every measured outcome is clinically relevant, even though data are heterogeneous and limited. This review is one of the few to synthesize long‐term, multidomain outcomes following.

However, important limitations should be acknowledged, including small sample sizes, outcome heterogeneity, overlapping cohorts and selective reporting. The evidence base is small and predominantly observational, with only one RCT identified by our literature search, limiting causal inference and increasing susceptibility to confounding. Outcomes were heterogeneous in definition, reporting and follow‐up duration. A major limitation of the current evidence base is the lack of standardized neurodevelopmental assessment tools, with most studies relying on informal screening rather than validated diagnostic instruments. Owing to the considerable heterogeneity in study design, outcome definitions, follow‐up duration, imaging modality and measurement techniques used, and the limited available data, meta‐analysis and formal assessment of publication bias could not be performed. The inability to formally assess publication bias means that reported outcomes may be influenced by selective reporting. Additionally, long‐term outcomes are reported almost exclusively in survivors, which introduces substantial survivorship bias and may lead to overestimation of favorable outcomes. We identified overlapping cohorts from two institutions and adjusted for this by counting each participant only once, although we cannot rule out additional unreported overlaps between studies from the same high‐volume centers. Temporal changes in FETO technique, selection criteria and neonatal care may limit comparability between early and contemporary studies. Not all of the included studies were designed primarily to assess long‐term outcome, resulting in incomplete or selective reporting across outcome domains. Finally, a broad inclusion strategy was adopted to capture the limited available evidence, but this meant that a standardized comparator group was not consistently available.

### Conclusions

Families should be counseled that survival following FETO for CDH does not equate necessarily to normal long‐term health, and that ongoing multidisciplinary follow‐up is essential. Further research with standardized long‐term outcome domains, alongside multicenter registries and well‐designed RCTs, are required to better define the long‐term impact of FETO. Long‐term morbidity remains common in infants who have undergone FETO but seems to improve over time and may be driven by factors other than fetal intervention, such as prenatal disease severity.

## Supporting information


**Appendix S1** Search strategy.
**Table S1** Excluded studies and reason for exclusion.
**Table S2** Imaging modalities used by included studies.
**Table S3** Long‐term infant outcomes reported by included studies.

## Data Availability

Research data are not shared.
