# Supplementary material for: Long‐term outcome after fetal endoscopic tracheal occlusion for congenital diaphragmatic hernia: systematic review
Source: Ultrasound Obstet Gynecol. 2026 Aug 29;68(5):613–20. doi: 10.1002/uog.70302 (PMC13730428; doi:10.1002/uog.70302)
Supplement: Supplementary file 1 [file UOG-68-613-s001.docx]

***Supporting information***

**Appendix S1** Search strategy

1) PubMed: ("Hernias, Diaphragmatic, Congenital"[Mesh] OR "congenital diaphragmatic hernia*" OR "CDH" OR "left diaphragmatic hernia*" OR "Bochdalek hernia*") AND ("Fetoscopy"[Mesh] OR "fetal endoscopic tracheal occlusion" OR "FETO" OR "fetoscopic tracheal occlusion" OR "fetoscopic endoluminal tracheal occlusion" OR "balloon occlusion")

Filters were applied to limit the search to the English language, human studies and exclusion of preprints

2) MEDLINE:

| 1. | exp Hernias, Diaphragmatic, Congenital/ |
| --- | --- |
| 2. | CDH.mp. [mp=title, book title, abstract, original title, name of substance word, subject heading word, floating sub-heading word, keyword heading word, organism supplementary concept word, protocol supplementary concept word, rare disease supplementary concept word, unique identifier, synonyms, population supplementary concept word, anatomy supplementary concept word] |
| 3. | congenital diaphragmatic hernia*.mp. [mp=title, book title, abstract, original title, name of substance word, subject heading word, floating sub-heading word, keyword heading word, organism supplementary concept word, protocol supplementary concept word, rare disease supplementary concept word, unique identifier, synonyms, population supplementary concept word, anatomy supplementary concept word] |
| 4. | left diaphragmatic hernia*.mp. [mp=title, book title, abstract, original title, name of substance word, subject heading word, floating sub-heading word, keyword heading word, organism supplementary concept word, protocol supplementary concept word, rare disease supplementary concept word, unique identifier, synonyms, population supplementary concept word, anatomy supplementary concept word] |
| 5. | Bochdalek hernia*.mp. [mp=title, book title, abstract, original title, name of substance word, subject heading word, floating sub-heading word, keyword heading word, organism supplementary concept word, protocol supplementary concept word, rare disease supplementary concept word, unique identifier, synonyms, population supplementary concept word, anatomy supplementary concept word] |
| 6. | 1 or 2 or 3 or 4 or 5 |
| 7. | exp Fetoscopy/ |
| 8. | Fetal endoscopic tracheal occlusion.mp. [mp=title, book title, abstract, original title, name of substance word, subject heading word, floating sub-heading word, keyword heading word, organism supplementary concept word, protocol supplementary concept word, rare disease supplementary concept word, unique identifier, synonyms, population supplementary concept word, anatomy supplementary concept word] |
| 9. | FETO.mp. [mp=title, book title, abstract, original title, name of substance word, subject heading word, floating sub-heading word, keyword heading word, organism supplementary concept word, protocol supplementary concept word, rare disease supplementary concept word, unique identifier, synonyms, population supplementary concept word, anatomy supplementary concept word] |
| 10. | fetoscopic tracheal occlusion.mp. [mp=title, book title, abstract, original title, name of substance word, subject heading word, floating sub-heading word, keyword heading word, organism supplementary concept word, protocol supplementary concept word, rare disease supplementary concept word, unique identifier, synonyms, population supplementary concept word, anatomy supplementary concept word] |
| 11. | fetoscopic endoluminal tracheal occlusion.mp. [mp=title, book title, abstract, original title, name of substance word, subject heading word, floating sub-heading word, keyword heading word, organism supplementary concept word, protocol supplementary concept word, rare disease supplementary concept word, unique identifier, synonyms, population supplementary concept word, anatomy supplementary concept word] |
| 12. | balloon occlusion.mp. [mp=title, book title, abstract, original title, name of substance word, subject heading word, floating sub-heading word, keyword heading word, organism supplementary concept word, protocol supplementary concept word, rare disease supplementary concept word, unique identifier, synonyms, population supplementary concept word, anatomy supplementary concept word] |
| 13. | 7 or 8 or 9 or 10 or 11 or 12 |
| 14. | 6 and 13 |
| 15. | limit 14 to (english language and humans and "remove preprint records") |

3) Embase

| 1. | exp Hernias, Diaphragmatic, Congenital/ |
| --- | --- |
| 2. | CDH.mp. [mp=title, abstract, heading word, drug trade name, original title, device manufacturer, drug manufacturer, device trade name, keyword heading word, floating subheading word, candidate term word] |
| 3. | congenital diaphragmatic hernia*.mp. [mp=title, abstract, heading word, drug trade name, original title, device manufacturer, drug manufacturer, device trade name, keyword heading word, floating subheading word, candidate term word] |
| 4. | left diaphragmatic hernia*.mp. [mp=title, abstract, heading word, drug trade name, original title, device manufacturer, drug manufacturer, device trade name, keyword heading word, floating subheading word, candidate term word] |
| 5. | Bochdalek hernia*.mp. [mp=title, abstract, heading word, drug trade name, original title, device manufacturer, drug manufacturer, device trade name, keyword heading word, floating subheading word, candidate term word] |
| 6. | 1 or 2 or 3 or 4 or 5 |
| 7. | exp Fetoscopy/ |
| 8. | Fetal endoscopic tracheal occlusion.mp. [mp=title, abstract, heading word, drug trade name, original title, device manufacturer, drug manufacturer, device trade name, keyword heading word, floating subheading word, candidate term word] |
| 9. | FETO.mp. [mp=title, abstract, heading word, drug trade name, original title, device manufacturer, drug manufacturer, device trade name, keyword heading word, floating subheading word, candidate term word] |
| 10. | fetoscopic tracheal occlusion.mp. [mp=title, abstract, heading word, drug trade name, original title, device manufacturer, drug manufacturer, device trade name, keyword heading word, floating subheading word, candidate term word] |
| 11. | fetoscopic endoluminal tracheal occlusion.mp. [mp=title, abstract, heading word, drug trade name, original title, device manufacturer, drug manufacturer, device trade name, keyword heading word, floating subheading word, candidate term word] |
| 12. | balloon occlusion.mp. [mp=title, abstract, heading word, drug trade name, original title, device manufacturer, drug manufacturer, device trade name, keyword heading word, floating subheading word, candidate term word] |
| 13. | 7 or 8 or 9 or 10 or 11 or 12 |
| 14. | 6 and 13 |
| 15. | limit 14 to (english language and humans and "remove preprint records") |

4) Cochrane CENTRAL

ID	Search

#1	MeSH descriptor: [Hernias, Diaphragmatic, Congenital] explode all trees

#2	MeSH descriptor: [Fetoscopy] explode all trees

#3	congenital diaphragmatic hernia*

#4	CDH

#5	left diaphragmatic hernia*

#6	Bochdalek hernia*

#7	#1 OR #3 OR #4 OR #5 OR #6

#8	fetal endoscopic tracheal occlusion

#9	FETO

#10	fetoscopic tracheal occlusion

#11	fetoscopic endoluminal tracheal occlusion

#12	balloon occlusion

#13	#2 OR #8 OR #9 OR #10 OR #11 OR #12

#14	#7 AND #13

Filters were applied to limit the search to the English language, human studies and exclusion of preprints

**Table S1** Excluded studies and reason for exclusion

| *No.* | *Study* | *Journal* | *Reason for exclusion* |
| --- | --- | --- | --- |
| 1 | Deprest (2004) | Ultrasound in Obstetrics and Gynecology | Long-term outcomes not reported |
| 2 | Debeer (2011) | Paediatric Respiratory Reviews | Conference abstract only |
| 3 | Verbelen (2012) | Interactive Cardiovascular and Thoracic Surgery | Conference abstract only |
| 4 | Verbelen (2013) | European Journal of Cardio-thoracic Surgery | Insufficient sample size |
| 5 | Proesmans (2015) | American Journal of Respiratory and Critical Care Medicine | Conference abstract only |
| 6 | Li (2016) | Journal of Pediatric Gastroenterology and Nutrition | Conference abstract only |
| 7 | Li (2016) | Journal of Pediatric Gastroenterology and Nutrition | Conference abstract only |
| 8 | Persico (2017) | Fetal Diagnosis and Therapy | Long-term outcomes not reported |
| 9 | Proesmans (2017) | European Respiratory Journal | Conference abstract only |
| 10 | Ruano (2017) | American Journal of Obstetrics and Gynecology | Conference abstract only |
| 11 | Snoek (2017) | Neonatology | Long-term outcomes not reported |
| 12 | Van Ginderdeuren (2017) | Neonatology | Outcomes not stratified by FETO exposure |
| 13 | Style (2018) | American Journal of Obstetrics and Gynecology | Conference abstract only |
| 14 | Russo (2019) | American Journal of Obstetrics and Gynecology | Conference abstract only |
| 15 | Amodeo (2020) | Frontiers in Pediatrics | Follow up < 1 year |
| 16 | Wada (2020) | Journal of Obstetrics and Gynaecology Research | Long-term outcomes not reported |
| 17 | Cordier (2021) | Ultrasound in Obstetrics and Gynecology | Outcomes not stratified by FETO exposure |
| 18 | Espinoza (2021) | Journal of Perinatal Medicine | Conference abstract only |
| 19 | Russo (2021) | Ultrasound in Obstetrics and Gynecology | Long-term outcomes not reported |
| 20 | Basurto (2023) | American Journal of Obstetrics and Gynecology | Conference abstract only |
| 21 | Van der Veeken (2023) | American Journal of Obstetrics and Gynecology | Conference abstract only |
| 22 | Eastwood (2024) | BJOG: An International Journal of Obstetrics and Gynaecology | Outcomes not stratified by FETO exposure |
| 23 | (2024) | Clinicaltrials.gov | Conference abstract only |

**Table S2** Imaging modalities used by included studies

| **Study** | Imaging modality |
| --- | --- |
| **Harrison (2003)^20^** | Ultrasound  *(Fetuses eligible for prenatal intervention met criteria including (1) diagnosis of CDH made before 25 weeks’ gestation, (2) a major portion of the liver herniated into the hemithorax, (3) lung-to-head ratio (LHR) less than 1.4 for left CDHs,1 (4) normal karyotype and echocardiogram, and (5) no other anomalies detected by prenatal ultrasonography.)* |
| **Cortes (2005)^12^** | Ultrasound  *(Fetuses identified with left CDH with liver herniated into the thorax and an LHR less than 1.4 were eligible for randomization if there were no other anomalies (by ultrasound and fetal echocardiogram)* |
| **Deprest (2005)^23^** | Ultrasound  *(Only patients with LHR measurements, obtained by experienced sonographers and with determined liver position by ultrasound (and in doubt by MRI), both 28 wks, were included.)* |
| **Ali (2013)^13^** | Not stated which method used  However, in clinical practice and in this context (King’s College Hospital fetal therapy program, selection for FETO), LHR is measured by prenatal ultrasound |
| **Zani (2014)^22^** | LHR not reported |
| **Belfort (2017)^24^** | Ultrasound  *(US-LHR (Pre-FETO) stated in Table 1)* |
| **Morandi (2018)^17^** | Ultrasound  *(The decision to perform the FETO procedure at our Fetal Surgery Center was taken according to the combined evaluation of the observed/expected lung-to-head ratio (O/E LHR), measured by ultrasound)* |
| **Ali (2019)^21^** | Not stated which method used However, in clinical practice and in this context (King’s College Hospital fetal therapy program, selection for FETO), LHR is measured by prenatal ultrasound  *(Fetuses were eligible for FETO if they had a left CDH and lung to head ratio (LHR)≤1.0 or a right-sided CDH with the liver in the chest)* |
| **Style (2019)^18^** | Prenatal magnetic resonance imaging (MRI) and Ultrasound  *(CDH cases were classified retrospectively into mild, moderate and severe groups based on prenatal magnetic resonance imaging indices (observed-to-expected total fetal lung volume and percentage of intrathoracic liver herniation). Lung-to-head ratio (LHR) and O/E-LHR obtained by ultrasound were also collected)* |
| **Macchini (2020)^25^** | Ultrasound  *(The indication to the FETO procedure at our Fetal Surgery Center was based on the combined evaluation of the O/E LHR, measured by ultrasound (20), and the side of the defect (left,right, and bilateral)* |
| **Sferra (2023)^14^** | Not stated which method used However, in clinical practice and in this context (Johns Hopkins fetal therapy program, selection for FETO), LHR is measured by prenatal ultrasound  *(Children with severe CDH were identified prenatally based on O/E LHR with evidence of intrathoracic liver herniation)* |
| **Pertierra Cortada (2023)^15^** | No stated which method used Since MRI is never mentioned and no MRI metrics are reported, this study is implicitly using ultrasound LHR  *(Prenatal information included CDH laterality, liver position, LHR, O/E LHR, and disease severity classification (in relation to liver position and LHR measurements)* |
| **Dütemeyer (2024)^19^** | Prenatal magnetic resonance imaging (MRI) |
| **Sferra (2024)^16^** | Not stated which method used However, in clinical practice and in this context (Johns Hopkins fetal therapy program, selection for FETO), LHR is measured by prenatal ultrasound  *(The inclusion criteria for the FETO cohort included prenatal findings of severe CDH, namely left or right sided CDH with an O/E LHR<30% with intrathoracic liver herniation)* |

**Table S3** Long-term infant outcome reported by included studies

| Long-term Outcomes | | | | | | | |
| --- | --- | --- | --- | --- | --- | --- | --- |
| Study | **Growth and nutritional** | **Neurodevelopmental** | **Audiological** | **Cardiac** | **Respiratory** | **Gastrointestinal** | **Musculoskeletal** |
| Harrison (2003)^20^ | NA | NA | NA | NA | - In the group of long-term survivors, 7 of 13 infants have had some form of tracheal problem.  - The 2 most significant have been bilateral recurrent laryngeal nerve injury and tracheomalacia. These were found at bronchoscopy when extubation of these infants was not possible.  - Four infants have had bilateral nerve injury leading to vocal cord paresis. Each had stridor shortly after extubation and required a tracheostomy for airway protection.  - Two have subsequently died from accidents caused by tracheostomy dislodgement at home.  - Two infants have had tracheomalacia with collapse of the airway on inspiration, necessitating stenting in both and a Cotton procedure in one with resection of a stenotic portion of trachea. - Two infants had pleural or lung infections | Significant gastroesophageal reflux in 7 patients; small bowel obstruction and necrotizing enterocolitis with sepsis developed in one other patient. | NA |
| Cortes (2005)^12^ | - Mean z values at both evaluations (except control, weight for-length [WFL] z score at 2 years) were negative, consistent with growth below the sex- and age-appropriate population means - Failure to thrive was prevalent at 1 year, although considerable catch-up growth occurred by 2 years (1 year: 86% [6/7]; 2 years: 34% [2/5]). - Proportion of patients requiring GER medications did not differ between groups at 1- and 2- year evaluations (total requiring at 1 year: 44% [7/16], P = .36; 2 years: 63% [10/16,] P = .63) - Growth failure was severe despite aggressive medical management. Fifty percent of the subjects were receiving GT feeds at 1 year and 44% were receiving GT feeds at 2 years (P = 1.0 and P = .62 at 1 and 2 years for differences between groups, respectively). | - No differences between groups in neuromotor, cognitive, or composite neurodevelopmental outcomes at 1 year or at 2 years (neuromotor, cognitive, composite neurodevelopmental outcome) | - Hearing loss requiring amplification has been detected in 7 of 16 patients with no differences between groups  - Abnormal neonatal brainstem audiometry: 1 (14%) - Abnormal or suspect at year 1: 0 (0%) - Abnormal or suspect at year 2: 1 (14%) - Amplification at year 3: 3 (43%) | No evidence of right ventricular hypertrophy on any echocardiogram, and there was good biventricular systolic function on all studies | - Of the 7 subjects who required oxygen supplementation at neonatal hospital discharge, 3/7 required it at the 1-year evaluation and 2/7 at the 2-year evaluation - 43% of the subjects were using pulmonary medications (diuretics, bronchodilators, and/or anti-inflammatory drugs) at the 1-year evaluation - Oxygen saturations on room air: groups at 1 year: 94.1% ± 4.7% or 2 years 95.8% ± 3.1% - Respiratory rate was somewhat elevated 40.7 ± 11.9 breaths/min; tidal volume was somewhat decreased (3.8 ± 1.7 mL/kg), and minute ventilation: 150 ± 70 mL/kg/min; Peak expiratory flows: 0.058 ± 0.031 L/cm | 43% of patients had recurrent herniations | NA |
| Deprest (2005)^23^ | NA | 10 long-term surviving babies are now aged 7 to 26 (median 19) months without known neurologic morbidity or developmental problems | NA | NA | NA | NA | NA |
| Ali (2013)^13^ | 7 (25% of survivors) had growth failure | 1 (1% of survivors) had delayed motor milestones | 2 (7.1% of survivors) had hearing loss | NA | 5 (18% of survivors) had reactive airways disease  6 (21% of survivors) had upper airway symptoms at follow-up | - 7 (25% of survivors) had hernia recurrence - 9 (33% of survivors) had antireflux medications - 4 (15% of survivors) had surgery for GO reflux | 10 (36% of survivors) had thoracic or spinal deformity |
| Zani (2014)^22^ | NA | NA | NA | NA | - On chest radiograph (CR) review, the trachea was wider at T1 (8.6 ± 0.3 mm vs 6.4 ± 0.2 mm; P < .0001) and at the widest tracheal point (WTP) (8.9 ± 0.4 mm vs 6.9 ± 0.3 mm; P < .0001) - There was no difference in tracheal width between survivors and nonsurvivors in the FETO group at all 3 points (T1: 8.4 ± 0.4 vs 9.4 ± 0.7 mm, P = .15; WTP: 8.8 ± 0.4 vs 9.5 ± 0.7 mm, P = .19; carina: 6.4 ± 0.2 vs 6.6 ± 0.5 mm, P = .60). - In both groups (FETO and non-FETO), tracheas grew progressively at a similar rate of increase (slope difference at T1, P = .38; at the WTP, P = .96; at the carina, P = .58) - Tracheal width was significantly larger on the last CR compared with the preoperative CR in the non-FETO group at all levels (T1, P = .02; WTP, P = .001; carina, P = .0001) and for the FETO group at the WTP (P < .0001) and the carina (P < .0001), but not at T1 (P = .12).  - In the FETO group, the WTP was most frequently at the level of T3 (42%) and less frequently at T2 (25%), T4 (17%), T1 (12%), or T5 (4%). - There was no difference in d1-BOI between the FETO and non-FETO groups. | - 2 patients in the FETO group (10%) underwent fundoplication for severe gastroesophageal reflux  - Diaphragmatic hernia recurred in 6 patients in the FETO group (30%) | 7 infants in the FETO group (35%) had significant chest wall deformities (P = .40), including pectus excavatum, pectus carinatum, and asymmetry |
| Belfort (2017)^24^ | NA | NA | NA | Extracorporeal membrane oxygenation utilization was also higher in the non-treated group Need for ECMO - FETO: 3/10 (30%) - No FETO: 7/9 (77.8%) | 2/11 needed supplemental oxygen to 1y and 2y of life | NA | NA |
| Morandi (2018)^17^ | NA | NA | NA | No differences between the two groups were observed in the rate of persistent pulmonary hypertension at 1 year of age (FETO: 1/13 patients, 7.7%; no FETO: 2/34 patients, 5.9%; p = 1) | Comparison of tracheal diameters: - T1: 12 months 6.14±0.33; 24 months 7.71±1.18; 36 months 8.44±0.46 - Carina: 12 months 6.89±0.64; 24 months 8.32±1.49; 36 months 9.80±1.13 - Maximal diameter: 12 months 9.17±0.61; 24 months 10.37±2.06; 36 months 12.82±0.68 No significant differences in PFT performed between 12 and 18 months of life were found between the two groups for all the analyzed parameters. | NA | NA |
| Ali (2019)^21^ | NA | NA | NA | NA | Chronic cough 14 (33%) for LCDH and 7 (70%) for RCDH | - Hernia recurrence 9 (21%) for LCDH and 4 (40%) for RCDH - Fundoplication 4 (9%) for LCDH and 2 (20%) for RCDH | Pectus deformity 17 (40%) for LCDH and 6 (60%) for RCDH. Scoliosis 5 (12%) for LCDH and 3 (30%) for RCDH |
| Style (2019)^18^ | NA | NA | NA | Only 28% (7/25) of the patients who had severe CDH in the non-FETO cohort showed resolution of pulmonary hypertension prior to 1 year of age, in contrast to 69% (11/16) of pulmonary hypertension resolution observed in infants in the FETO group | NA | NA | NA |
| Macchini (2020)^25^ | NA | NA | NA | NA | - Maximal tracheal caliber 10±2.09 mm - Lung total volume 337±46.7 (ml) - Respiratory rate 33.9±11.5 (breaths/min) | - GERD symptoms 5/10 - Age at pH-metry (months) 20.9 - Weight at pH-metry (kg) 9.2±1 - Height at pH-metry (cm) 74.9 ± 4.8 - Reflux index (%) 4.95 - Acid GER/24h(num.) 69.5 - Acid GER >5min(num.) 1.04 | NA |
| Sferra (2023)^14^ | Erythromycin use in 4/12 (33% of patients)  Feeding at most-recent follow-up:  - PO 3/12 (25%)  - Nasogastric/nasoduodenal tube 1/12 (8%)  - Gastrostomy/jejunostomy 8/12 (67%) | Anti-epileptic medication use in 0 patients | NA | NA | - Sildenafil use decreased by 29% in FETO patients and 34% in non-FETO patients (p = 0.53). - Patients in the FETO cohort decreased their bronchodilator/inhaled corticosteroid use by 21%, whereas patients in the non-FETO cohort increased their use by 7% (p = 0.70).  - The number of patients requiring tracheostomy or supplemental oxygen decreased in both cohorts.   - Sildenafil use in 5/12 (42% of patients) - Bosentan use in 2/12 (17% of patients) - Treprostinil use in 0 patients - Bronchodilator/inhaled corticosteroids use in 7/12 (58% of patients) - Supplemental oxygen use in 1/12 (8% of patients)  - Tracheostomy in 1/12 (8% of patients) | - Number gastrointestinal medications at most-recent follow-up: 0 (0-1) (median, IQR) - Antacid 5/12 (42% of patients) - Antacid use decreased in both cohorts (p = 0.35).  - There was a 12% decrease in ≥50% gastrostomy/jejunostomy feeds among FETO patients, but a 33% increase in non-FETO patients. - Nearly identical gastrostomy/jejunostomy rates at the most recent follow-up (67% for FETO) | NA |
| Pertierra Cortada (2023)^15^ | Analyzing failure to thrive, no differences were detected when prenatal severity or FETO treatment (p=0.731) was considered. Failure to thrive at 2y moderate prenatal classification: 0.66 (0.11–3.7), relation expressed in OR (p value) | - Abnormal neurodevelopment at 2y moderate prenatal classification: 2.64 (0.26–26), relation expressed in OR (p value) - No differences in neurological outcomes in relation to prenatal severity assessment Fig. 6A), nor between FETO and non-FETO populations | NA | NA | - The prevalence of respiratory problems at 2 years was higher in the FETO population (OR: 3.4; p=0.009) even when prenatal severity correction was applied to the non-FETO population (OR: 2.5; p=0.027) - Respiratory problems at 2y moderate prenatal classification: 1.25 (0.27–5.7), relation expressed in OR (p value) | - There was a GERD prevalence of 37.50% in the FETO population. - Gastroesophagic reflux disease at 2y moderate prenatal classification: 1.61 (0.32–7.9), relation expressed in OR (p value) - No differences were found between FETO and non FETO populations for oral aversion (p=0.681). | NA |
| Dütemeyer (2024)^19^ | 6 had feeding by nasogastric tube or gastro- or jejunostomy at 1 year of life and 3 had feeding by nasogastric tube or gastro- or jejunostomy at 2 years of life | 1 had brain lesion at 2 years of life 12 had regular school at 6 years of life | NA | 1 patient had persistent pulmonary hypertension at 1 year of life | 3 patients had Oxygen therapy by nasal canula or tracheostomy at 1 and 2 years of life | NA | NA |
| Sferra (2024)^16^ | NA | Capute Scales - CLAMS: 12 months 80.1 (67.6-86.7); >24 months 92.3 (74-98.6) - CAT-DQ: 12-months 81.3 (62.1-89.4); >24 months 90.8 (80-101.4) | NA | NA | NA | NA | NA |

Only first author is given for each study. Not all outcomes were reported at all timepoints.
